# Effects of gut microbiota-targeted interventions on pain, physical function, and inflammatory biomarkers in osteoarthritis: a systematic review and meta-analysis of randomized controlled trials

**DOI:** 10.3389/fcimb.2026.1907672

**Published:** 2026-09-04

**Authors:** Tao Li, Juncheng Long, Yazhong Zhang, HongMei Luo, JianPing Yu

**Affiliations:** 1 The Affiliated Hospital of Jiangxi University of Chinese Medicine Rheumatology Department, Nanchang, Jiangxi, China; 2 Institute of Traditional Chinese Medicine Health Industry, China Academy of Chinese Medical Sciences, Nanchang, China; 3 Wuzhou Hospital of Traditional Chinese Medicine Department of Traumatic Orthopedics, Wuzhou, Guangxi, China

**Keywords:** gut microbiota, gut-joint axis, meta-analysis, osteoarthritis, prebiotics, probiotics, systematic review

## Abstract

**Background:**

Gut microbiota dysbiosis has been implicated in osteoarthritis (OA) through the gut–joint axis, but the clinical efficacy of gut microbiota-targeted interventions remains unclear. This systematic review and meta-analysis evaluated the effects of these interventions on clinical outcomes and inflammatory biomarkers in OA.

**Methods:**

PubMed, Embase, Web of Science, CNKI, and Wanfang were searched from inception to 20 January 2026 for randomized controlled trials (RCTs) of probiotics, prebiotics, synbiotics, or dietary modification in adults with OA. Primary outcomes were visual analog scale (VAS) pain, Western Ontario and McMaster Universities Osteoarthritis Index (WOMAC) pain, WOMAC function, and WOMAC total score; secondary outcomes included WOMAC stiffness, body weight, knee flexion angle, cartilage oligomeric matrix protein (COMP), high-sensitivity C-reactive protein (hs-CRP), erythrocyte sedimentation rate (ESR), interleukin (IL)-1β, and IL-6. Standardized mean differences (SMDs) with 95% CIs were pooled using random-effects models; risk of bias was assessed with RoB 2.0. Subgroup analyses, univariable meta-regression, trial sequential analysis (TSA), and GRADE were used to grade certainty.

**Results:**

Eighteen RCTs (2,080 participants) were included. Interventions reduced VAS pain (SMD = −0.66, 95% CI: −1.27 to −0.06, *P* = 0.03), WOMAC pain (SMD = −0.62, 95% CI: −0.97 to −0.27, *P* = 0.004), WOMAC function (SMD = −0.40, 95% CI: −0.64 to −0.17, *P* = 0.0006), and WOMAC total score (SMD = −0.78, 95% CI: −1.26 to −0.30, *P* = 0.009) and lowered hs-CRP, ESR, and IL-1β. WOMAC stiffness, WOMAC physical function, body weight, knee flexion angle, COMP, and IL-6 did not differ significantly. All 18 trials were rated “some concerns” overall on RoB 2.0. Probiotics showed larger effects than dietary interventions, with appreciable residual heterogeneity. Information accrual reached the required size only for WOMAC pain and WOMAC function; VAS pain, WOMAC total score, and hs-CRP were promising but not yet conclusive. GRADE certainty ranged from high (WOMAC function) to very low (VAS pain); unchanged COMP indicates no support for cartilage protection or disease modification.

**Conclusion:**

Gut microbiota-targeted interventions, particularly probiotics, may improve pain, physical function, and systemic inflammation in OA. The evidence is compatible with symptomatic benefit but does not support cartilage protection or disease modification. Because certainty ranges from high to very low and information accrual is incomplete for most outcomes, these findings are provisional pending larger standardized RCTs.

**Systematic review registration:**

https://www.crd.york.ac.uk/PROSPERO/home, identifier CRD42023472181.

## 1 Introduction

Osteoarthritis (OA) is the most prevalent chronic joint disease worldwide, affecting over 528 million individuals (Long et al., 2022; GBD 2021 Osteoarthritis Collaborators, 2023). Current pharmacological therapies, including NSAIDs, acetaminophen, and intra-articular corticosteroids, remain largely symptomatic and carry dose-limiting adverse effects with long-term use, while joint replacement is reserved for end-stage disease (Kolasinski et al., 2020; Arden et al., 2021; da Costa et al., 2017; Price et al., 2018). This therapeutic gap has driven interest in the emerging “gut–joint axis,” whereby intestinal dysbiosis promotes OA pathogenesis through increased intestinal permeability, systemic translocation of lipopolysaccharide (LPS), activation of TLR4/NF-κB signaling, and release of pro-inflammatory cytokines [interleukin (IL)-1β, IL-6, tumor necrosis factor alpha (TNF-α)] in joint tissues (Boer et al., 2019; Guido et al., 2021; Hao et al., 2021). Modulation of the gut microbiome through probiotics, prebiotics, synbiotics, and dietary modifications has therefore been explored as a potential therapeutic strategy (Cani, 2018; Biver et al., 2019; de Sire et al., 2020). However, existing systematic reviews have primarily characterized microbiota composition changes rather than evaluating clinical interventional outcomes (Bonato et al., 2022; Moyseos et al., 2024), and the optimal intervention type, strain, dosage, and duration remain poorly defined (Schott et al., 2018). The present systematic review and meta-analysis aims to comprehensively synthesize evidence from RCTs evaluating gut microbiota-targeted interventions on pain, physical function, and inflammatory biomarkers in OA patients and to provide a differentiated assessment of evidence certainty through subgroup analyses, meta-regression, trial sequential analysis (TSA), and grading of recommendations, assessment, development, and evaluation (GRADE) quality evaluation (Page et al., 2021).

## 2 Methods

### 2.1 Study design and registration

This systematic review and meta-analysis was conducted in accordance with the PRISMA 2020 guidelines and the Cochrane Handbook for Systematic Reviews of Interventions. The review protocol was prospectively registered in PROSPERO (registration number: CRD42023472181). Any deviations from the registered protocol are documented in the **Supplementary Materials**.

### 2.2 Eligibility criteria

#### 2.2.1 Inclusion criteria

Studies were eligible if they met the following PICOS criteria: (1) population: adult patients (aged ≥18 years) with clinically and/or radiographically diagnosed OA (ACR criteria or Kellgren–Lawrence grading), regardless of affected joint; (2) intervention: any gut microbiota-targeted intervention, including probiotics (single-strain or multistrain), prebiotics, synbiotics, postbiotics, fecal microbiota transplantation, or dietary interventions designed to modulate gut microbiota; (3) comparator: placebo, no treatment, or standard care; (4) outcomes: at least one predefined primary or secondary outcome; and (5) study design: randomized controlled trials.

#### 2.2.2 Exclusion criteria

Studies were excluded if they (1) included patients with other inflammatory arthritides unless OA patients were reported separately; (2) investigated non-gut microbiomes without gut microbiota-targeted intervention; (3) were non-randomized, observational, or non-primary studies; (4) used animal models exclusively; (5) did not report predefined outcomes; (6) were published in languages other than English or Chinese; and (7) had insufficient data with no author response.

### 2.3 Information sources and search strategy

A comprehensive search was conducted across PubMed, Embase, Web of Science, CNKI, and Wanfang databases from inception to 20 January 2026. The search strategy combined MeSH terms and free-text keywords for three core concepts: osteoarthritis, gut microbiota, and microbiota-targeted interventions. Supplementary methods included reference list screening, forward citation tracking, clinical trial registry searches (ClinicalTrials.gov, ICTRP, ChiCTR), and author contact.

### 2.4 Study selection

Records were imported into EndNote X9 for deduplication. Two reviewers (LT and LJC) independently screened titles/abstracts, then assessed full texts against eligibility criteria. Disagreements were resolved by discussion or consultation with a third reviewer.

### 2.5 Data extraction

Two reviewers independently extracted data using a standardized form, including study characteristics, participant demographics, intervention details (type, strain, CFU, dose, frequency, duration), comparator type, outcome data, and adverse events.

### 2.6 Outcome measures

**Figure 1:**
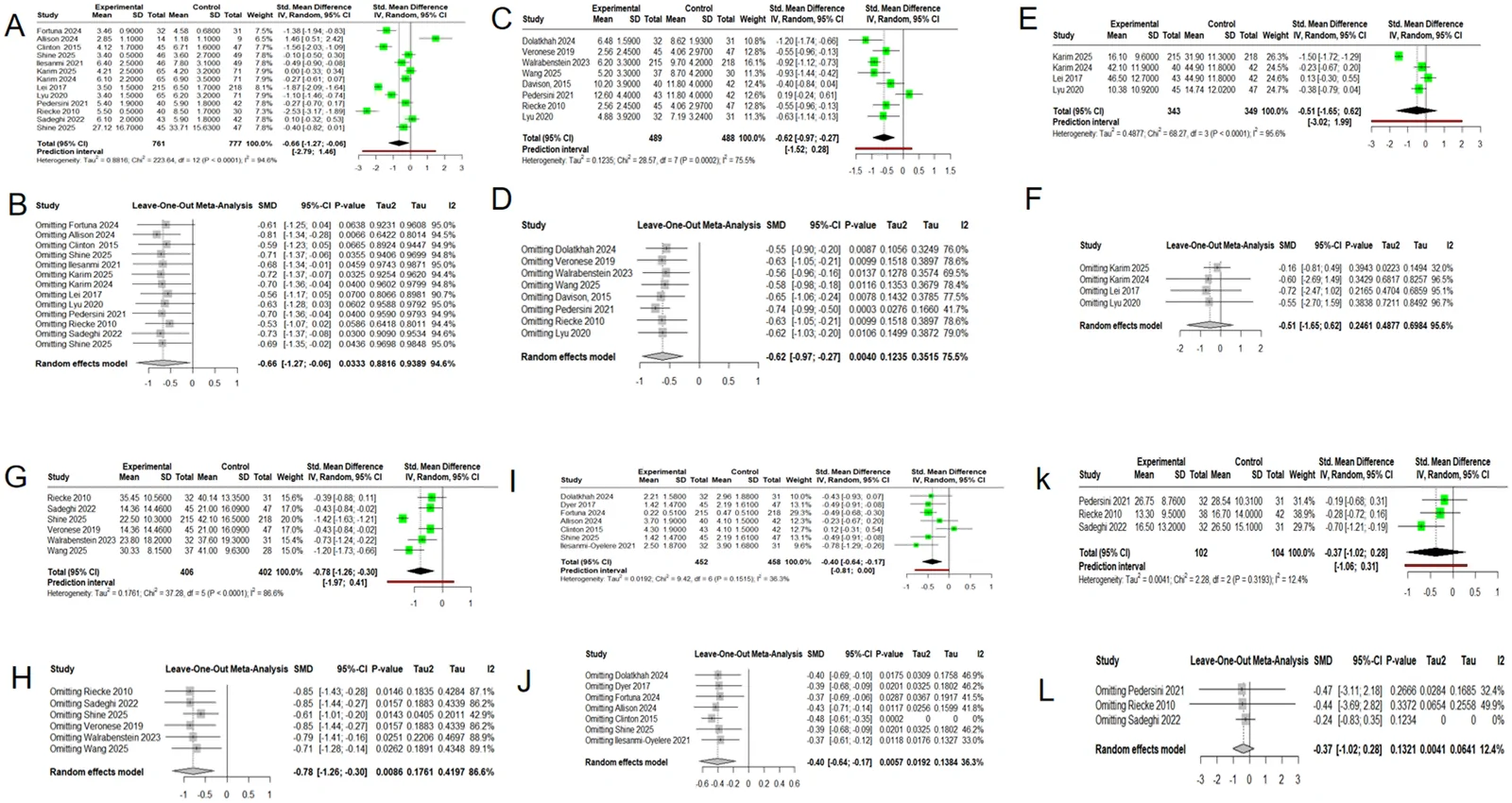
Forest plots and leave-one-out sensitivity analyses of gut microbiota-targeted interventions on pain and functional outcomes in osteoarthritis. **(A, B)** VAS pain (13 comparisons from 12 trials; Shine 2025 contributes two comparisons and therefore appears twice); **(C, D)** WOMAC pain subscale (*k* = 8); **(E, F)** WOMAC stiffness subscale (*k* = 4); **(G, H)** WOMAC total score (*k* = 6); **(I, J)** WOMAC function, primary functional analysis (*k* = 7); **(K, L)** WOMAC physical function, separate analysis of three trials (*k* = 3). Odd-numbered panels show forest plots of SMD with 95% CIs using random-effects models; even-numbered panels show corresponding leave-one-out sensitivity analyses.

Primary outcomes were visual analog scale (VAS) pain (0–100 mm or 0–10 cm), the Western Ontario and McMaster Universities Osteoarthritis Index (WOMAC) pain subscale, WOMAC function, and the WOMAC total score. Secondary outcomes included WOMAC stiffness, WOMAC physical function, body weight, knee flexion angle, cartilage oligomeric matrix protein (COMP), high-sensitivity C-reactive protein (hs-CRP), erythrocyte sedimentation rate (ESR), IL-1β, IL-6, and adverse events. Functional data were contributed by 10 trials in two non-overlapping sets, which were pooled separately and are reported under fixed and distinct labels throughout. “WOMAC function” denotes the primary functional analysis of seven trials (Clinton et al., 2015, Dyer et al., 2017, Ilesanmi-Oyelere 2021; Allison et al., 2024, Dolatkhah et al., 2024, Fortuna et al., 2024, and Shine et al., 2025), marked WOMAC-Func in **Table 1** and plotted in **Figures 1I,J**. “WOMAC physical function” denotes the separate analysis of the remaining three trials (Riecke et al., 2010; Pedersini et al., 2021, and Sadeghi et al., 2022), marked WOMAC-PF in **Table 1** and plotted in **Figures 1K,L**. The two analyses share no trials and are not combined at any point. Neither label is used interchangeably with the other, and the unqualified term “physical function” appears only in the general sense of the clinical domain, never as the name of either analysis.

**Table 1:** Characteristics of included randomized controlled trials, ordered by year of publication.

Study	Country/region	Design	Sample size (I/C)	Age, mean ± SD (I/C)	Joint site; KL grade	Intervention type	Specific composition (strain, dose, diet)	Frequency and duration	Comparator	Follow-up	Outcomes contributed
Riecke et al., 2010	Denmark	RCT, open-label	43/43	59.1 ± 9.5/59.1 ± 9.5	Knee OA; KL ≥ 2	Dietary	Low-energy diet (810 kcal/day) vs. very-low-energy diet (415 kcal/day)	Daily; 8 weeks + 8 weeks	Low-energy diet (active control)	16 weeks	VAS, WOMAC-Pain, WOMAC-Total, WOMAC-PF, BW, KFA, IL-1β
Clinton et al., 2015	USA	RCT, open-label	45/47	56.1 ± 8.4/60.0 ± 6.3	Generalized OA; –	Dietary	Whole-foods, plant-based diet (6-week elimination phase)	Daily; 6 weeks	Usual diet	6 weeks	VAS, WOMAC-Func, hs-CRP
Davison et al., 2015	UK	RCT, open-label	32/25	63 ± 6.0/62 ± 7.0	Generalized OA; –	Dietary	Mediterranean diet (counselling + food provision)	Daily; 16 weeks	Usual diet	16 weeks	WOMAC-Pain, COMP
Dyer et al., 2017	UK	RCT, open-label	32/31	66 ± 11/60 ± 12	Generalized OA; –	Dietary	Mediterranean diet (counselling)	Daily; 16 weeks	Usual diet/standard care	16 weeks	WOMAC-Func, hs-CRP, ESR, IL-6
Lei et al., 2017	Taiwan, China	RCT, double-blind	215/218	65.1 ± 9.3/60.8 ± 12.2	Knee OA; KL 1–3	Probiotic	*Lactobacillus casei* Shirota; 6.5 × 10^9^ CFU/day	Daily; 12 weeks	Placebo (matched capsule)	12 weeks	VAS, WOMAC-Stiff, BW
Veronese et al., 2019	Italy	RCT, open-label	45/47	61.1 ± 9.2/60.5 ± 8.8	Knee OA; KL ≥ 2	Dietary	Mediterranean diet (adherence score–driven)	Daily; 4 years	Non-Mediterranean diet	4 years	WOMAC-Pain, WOMAC-Total
Lyu et al., 2020	Taiwan, China	RCT, double-blind	65/71	Range 50–90	Knee OA; KL 1–3	Probiotic	*Streptococcus thermophilus* TCI633 (oral capsule); 3 × 10¹^0^ CFU/day	Daily; 6 weeks	Placebo (matched capsule)	6 weeks	VAS, WOMAC-Pain, WOMAC-Stiff, BW, IL-1β
Ilesanmi-Oyelere 2021	New Zealand	RCT, double-blind	65/71	>60 (range)	Generalized OA; –	Synbiotic	*Lactobacillus rhamnosus* HN001 + oligofructose-enriched inulin; 6.5 × 10^9^ CFU/day	Daily; 12 weeks	Placebo/standard care	12 weeks	VAS, WOMAC-Func, hs-CRP
Pedersini et al., 2021	Italy	RCT, double-blind	96/96	62.5 ± 6.4/63.1 ± 7.2	Knee OA; KL ≥ 2	Probiotic	Vivomixx multi-strain formulation (commercial product); 4.5 × 10¹¹ CFU/day	Daily; 16 weeks	Placebo	16 weeks	VAS, WOMAC-Pain, WOMAC-PF, BW, KFA
Sadeghi et al., 2022	Iran	RCT, feeding trial	45/47	54.6 ± 8.3/55.2 ± 7.9	Knee OA; KL ≥ 2	Dietary	Mediterranean diet vs. low-fat diet	Daily; 12 weeks	Low-fat diet (active control)	12 weeks	VAS, WOMAC-Total, WOMAC-PF, BW, KFA, IL-1β
Walrabenstein et al., 2023	Netherlands	RCT, open-label	32/32	63 ± 6.0/63 ± 5.8	Generalized OA; –	Dietary	“Plants for Joints” lifestyle program (plant-based diet + supplements + exercise)	Daily; 16 weeks	Usual care	16 weeks	WOMAC-Pain, WOMAC-Total, COMP
Allison et al., 2024	Australia	RCT, assessor-blind	42/46	60.5 ± 7.0/60.0 ± 8.3	Knee OA; KL ≥ 2	Dietary	Very-low-energy diet (<800 kcal/day) + exercise	Daily; 6 months	Exercise alone	6 months	VAS, WOMAC-Func
Dolatkhah et al., 2024	Iran	RCT, triple-blind	35/35 (32/31)	56.70 ± 9.05/54.37 ± 8.77	Knee OA; KL 2–3	Probiotic	*Saccharomyces boulardii* CNCM I-745; 1 × 10¹^0^ CFU/day	Daily; 12 weeks	Placebo (matched capsule)	12 weeks	WOMAC-Pain, WOMAC-Func, ESR, IL-6
Fortuna et al., 2024	Canada	RCT, double-blind	32/24	59.2 ± 9.7/59.1 ± 11.5	Knee OA; KL 2–3	Prebiotic	Oligofructose-enriched inulin (Orafti Synergy1); 16 g/day	Daily; 6 months	Placebo (maltodextrin)	6 months	VAS, WOMAC-Func, ESR, IL-6
Karim et al., 2024	UAE	RCT, double-blind	65/71	57.8 ± 9.4/56.2 ± 8.7	Knee OA; KL ≥ 2	Probiotic	Multi-strain probiotic capsule; total ≥ 1 × 10^9^ CFU/day	Daily; 6 months	Placebo	6 months	VAS, WOMAC-Stiff, BW, hs-CRP
Shine et al., 2025	Republic of Korea	RCT, double-blind	50/50	62.79 ± 6.91/63.15 ± 7.24	Knee OA; KL 1–2	Probiotic	*Latilactobacillus sakei* LB-P12; 5 × 10^9^ CFU/day	Once daily; 12 weeks	Placebo (matched capsule)	12 weeks	VAS (two comparisons), WOMAC-Func, WOMAC-Total, hs-CRP
Karim et al., 2025	UAE; Pakistan	RCT, double-blind	65/71	69.3 ± 5.5/67.3 ± 4.5	Knee OA; KL ≥ 2	Probiotic	Vivomixx multi-strain probiotic (8 strains); 4.5 × 10¹¹ CFU/day	Daily; 16 weeks	Placebo	16 weeks	VAS, WOMAC-Stiff, hs-CRP
Wang et al., 2025	China	RCT, open-label	45/47	Range 50–70	Knee OA; KL ≥ 2	Probiotic	*Bifidobacterium animalis* subsp. *lactis* Probio-M8 + chondroitin; 5 × 10¹^0^ CFU/day	Daily; 3 months	Chondroitin alone or placebo	3 months	WOMAC-Pain, WOMAC-Total, COMP

BW, body weight; CFU, colony-forming units; COMP, cartilage oligomeric matrix protein; ESR, erythrocyte sedimentation rate; hs-CRP, high-sensitivity C-reactive protein; I/C, intervention/control; IL, interleukin; KFA, knee flexion angle; KL, Kellgren–Lawrence grade; NR, not reported; OA, osteoarthritis; RCT, randomized controlled trial; VAS, visual analog scale; WOMAC, Western Ontario and McMaster Universities Osteoarthritis Index; WOMAC-Func, WOMAC function (primary functional analysis, Section 3.3.3); WOMAC-PF, WOMAC physical function (separate analysis, Section 3.3.6); WOMAC-Stiff, WOMAC stiffness subscale. Sample sizes are those randomized; the numbers analyzed for each outcome are given in the corresponding forest plot and may be smaller owing to attrition or incomplete outcome reporting. Trials contributing to each pooled analysis were as follows. VAS pain (*k* = 13 comparisons from 12 trials): Riecke et al., 2010; Clinton et al., 2015; Lei et al., 2017; Lyu et al., 2020; Ilesanmi-Oyelere 2021; Pedersini et al., 2021; Sadeghi et al., 2022; Allison et al., 2024; Fortuna et al., 2024; Karim et al., 2024; Shine et al., 2025 (two comparisons), and Karim et al., 2025. WOMAC pain (*k* = 8): Riecke et al., 2010; Davison et al., 2015; Veronese et al., 2019; Lyu et al., 2020; Pedersini et al., 2021; Walrabenstein et al., 2023; Dolatkhah et al., 2024, and Wang et al., 2025. WOMAC function (*k* = 7): Clinton et al., 2015; Dyer et al., 2017; Ilesanmi-Oyelere 2021; Allison et al., 2024; Dolatkhah et al., 2024; Fortuna et al., 2024, and Shine et al., 2025. WOMAC total (*k* = 6): Riecke et al., 2010; Veronese et al., 2019; Sadeghi et al., 2022; Walrabenstein et al., 2023; Shine et al., 2025, and Wang et al., 2025. WOMAC stiffness (*k* = 4): Lei et al., 2017; Lyu et al., 2020; Karim et al., 2024, 2025. WOMAC physical function (*k* = 3): Riecke et al., 2010; Pedersini et al., 2021; Sadeghi et al., 2022. Body weight (*k* = 6): Riecke et al., 2010; Lei et al., 2017; Lyu et al., 2020; Pedersini et al., 2021; Sadeghi et al., 2022; Karim et al., 2024. Knee flexion angle (*k* = 3): Riecke et al., 2010; Pedersini et al., 2021; Sadeghi et al., 2022. COMP (*k* = 3): Davison et al., 2015; Walrabenstein et al., 2023; Wang et al., 2025. hs-CRP (*k* = 6): Clinton et al., 2015; Dyer et al., 2017; Ilesanmi-Oyelere 2021; Karim et al., 2024; Shine et al., 2025; Karim et al., 2025. ESR (*k* = 3) and IL-6 (*k* = 3): Dyer et al., 2017; Dolatkhah et al., 2024; Fortuna et al., 2024. IL-1β (*k* = 3): Riecke et al., 2010; Lyu et al., 2020; Sadeghi et al., 2022.

### 2.7 Risk of bias assessment

Risk of bias was independently assessed by two reviewers using the revised Cochrane Risk of Bias tool (RoB 2.0), evaluating five domains: randomization process, deviations from intended interventions, missing outcome data, measurement of the outcome, and selection of reported results. Each domain was judged as “low risk of bias,” “some concerns,” or “high risk of bias.” Risk of bias figures were generated using robvis.

### 2.8 Quality of evidence assessment

The certainty of evidence for each outcome was evaluated using the GRADE approach (Guyatt et al., 2008). Starting from a “high” rating for RCT evidence, the certainty was downgraded based on five domains: risk of bias, inconsistency (*I*^2^ statistic and overlap of confidence intervals), indirectness, imprecision (width of 95% CI, total sample size relative to optimal information size, and TSA results including whether accrued information reached DARIS), and publication bias (funnel plot asymmetry and Egger’s test where applicable). The certainty was rated as high, moderate, low, or very low. A summary of the findings is presented in **Table 2**.

**Table 2:** GRADE evidence quality summary for primary and key secondary outcomes.

Outcome	Studies (k)	Participants	SMD (95% CI)	Risk of bias	Inconsistency	Indirectness	Imprecision	Publication bias	GRADE certainty
VAS pain	13	1,538	−0.66 [−1.27, −0.06]*	Not serious	Serious (−1)	Not serious	Serious (−1)	Serious (−1)	⊕○○○ very low
WOMAC pain	8	977	−0.62 [−0.97, −0.27]**	Not serious	Serious (−1)	Not serious	Not serious	Not assessed	⊕⊕⊕○ moderate
WOMAC function	7	910	−0.40 [−0.64, −0.17]***	Not serious	Not serious	Not serious	Not serious	Not assessed	⊕⊕⊕⊕ high
WOMAC total	6	808	−0.78 [−1.26, −0.30]**	Not serious	Serious (−1)	Not serious	Serious (−1)	Not assessed	⊕⊕○○ low
hs-CRP	6	855	−0.78 [−1.33, −0.23]*	Not serious	Serious (−1)	Not serious	Serious (−1)	Not assessed	⊕⊕○○ low
ESR	3	247	−0.47 [−0.64, −0.30]**	Not serious	Not serious	Not serious	Serious (−1)	Not assessed	⊕⊕⊕○ moderate
IL-1β	3	249	−0.36 [−0.58, −0.14]*	Not serious	Not serious	Not serious	Serious (−1)	Not assessed	⊕⊕⊕○ moderate
IL-6	3	213	0.09 [−0.45, 0.63] NS	Not serious	Not serious	Not serious	Serious (−1)	Not assessed	⊕⊕⊕○ moderate

GRADE symbols: ⊕⊕⊕⊕ = high; ⊕⊕⊕○ = moderate; ⊕⊕○○ = low; ⊕○○○ = very low. Imprecision incorporated TSA-DARIS results. For VAS pain, *k* denotes 13 comparisons contributed by 12 trials, with Shine 2025 providing two. Publication bias was formally tested (Egger’s test) only for VAS pain, the sole outcome with ≥10 contributing comparisons; for all the other outcomes, the domain is reported as “not assessed” because fewer than 10 studies contributed, and publication bias therefore cannot be excluded. Risk of bias was not downgraded because the RoB 2.0 judgement of “some concerns” for every trial arose from incomplete reporting of attrition and of pre-specified analyses rather than from features expected to bias the effect estimates; this judgement is discussed in Section 4.6.Abbreviations: CI, confidence interval; ESR, erythrocyte sedimentation rate; GRADE, Grading of Recommendations Assessment, Development and Evaluation; hs-CRP, high-sensitivity C-reactive protein; IL, interleukin; NS, not significant; SMD, standardized mean difference; VAS, visual analog scale; WOMAC, Western Ontario and McMaster Universities Osteoarthritis Index.

*P < 0.05; **P < 0.01; ***P < 0.001.

### 2.9 Data synthesis and statistical analysis

Continuous outcomes were pooled using SMD with Hedges’ *g* correction and 95% CIs using random-effects models (DerSimonian–Laird). Where a trial provided more than one eligible comparison for the same outcome, each comparison was entered separately; *k* therefore denotes the number of pooled comparisons, and the number of contributing trials is stated whenever it differs from *k*. Heterogeneity was assessed using Cochran’s *Q* test and *I*^2^ statistic. Sensitivity analyses used the leave-one-out method. Publication bias was assessed by funnel plot and Egger’s test when ≥10 studies were available.

Pre-specified subgroup analyses were conducted by intervention type, joint site, treatment duration, and geographic region. Tests for subgroup differences used the between-subgroup *Q* test (*P*_interaction_ < 0.10). Univariable random-effects meta-regression with REML estimation and Knapp–Hartung adjustment explored potential moderators of VAS pain effect size.

Trial sequential analysis (TSA) was performed to control for random error and to assess whether the cumulative evidence was conclusive (Wetterslev et al., 2017), using TSA software version 0.9.5.10 Beta (Copenhagen Trial Unit). DARIS was calculated with *α* = 0.05 (two-sided), power = 80%, and outcome-specific MCID based on OARSI-OMERACT consensus thresholds, using model-variance-based heterogeneity correction. Cumulative *Z*-curves were evaluated against O’Brien–Fleming monitoring boundaries and futility boundaries. Outcomes were assigned to one of three mutually exclusive categories: “sufficient information accrued” (the *Z*-curve crossed the monitoring boundary and the accrued information size reached or exceeded DARIS), “promising but not yet conclusive” (the pooled estimate was conventionally significant but the *Z*-curve did not cross the monitoring boundary and/or accrued information remained below DARIS), or “futility/stable null” (the *Z*-curve crossed the futility boundary). Because DARIS depends on the assumed MCID and on the heterogeneity correction applied, all TSA classifications are reported as provisional, and the descriptors “firm,” “conclusive,” and “definitive” are not applied to any outcome in this report. All analyses used RevMan 5.4 and R 4.2 (meta and metafor packages). *P* < 0.05 (two-sided) was considered significant.

## 3 Results

### 3.1 Study selection and characteristics

**Figure 2:**
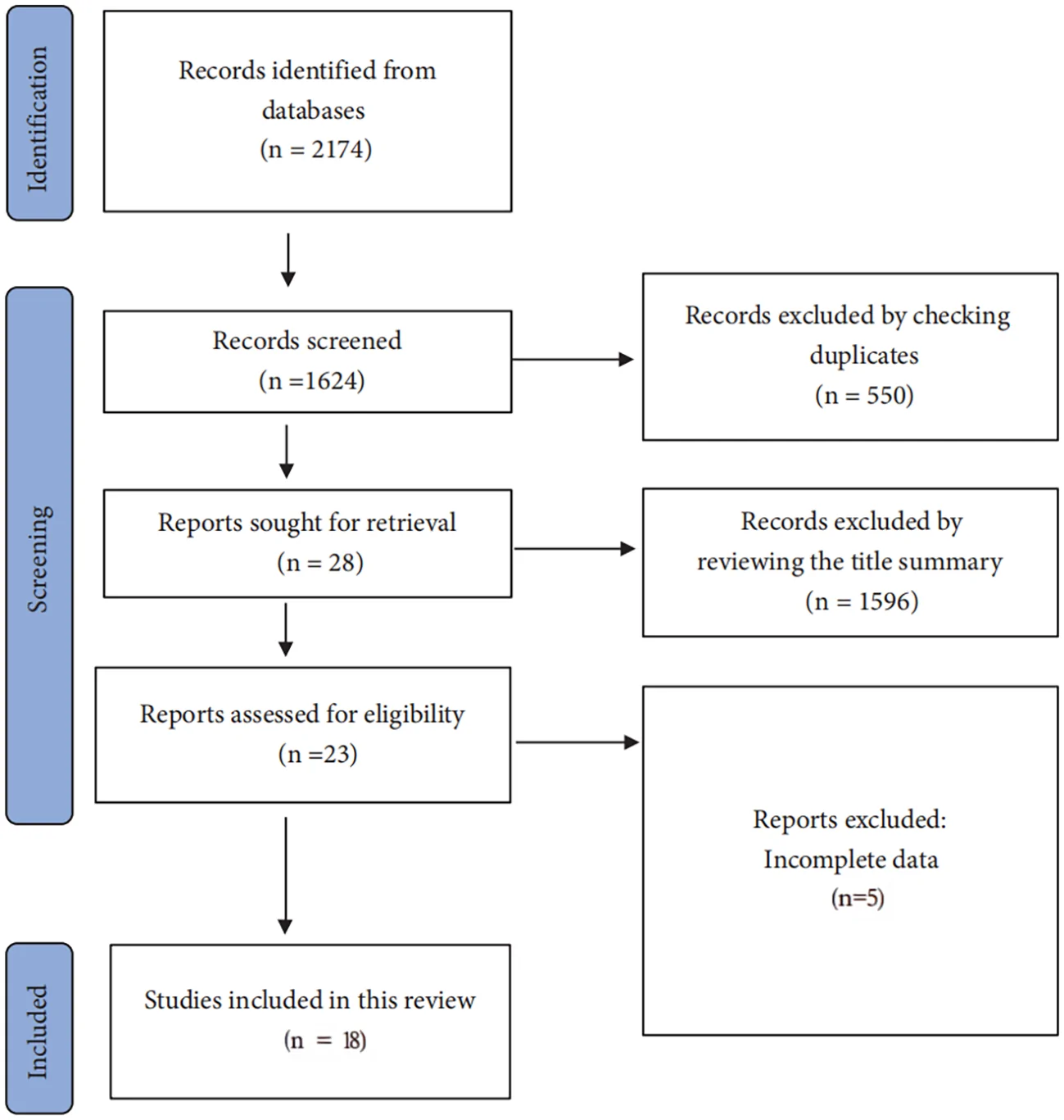
PRISMA 2020 flow diagram of the study selection process. The diagram summarizes the number of records identified through database searching (*n* = 2,174), records remaining after duplicate removal, records screened by title and abstract, full-text articles assessed for eligibility (*n* = 23), and the final number of studies included in the systematic review and meta-analysis (*n* = 18), together with the reasons for exclusion at each stage.

The initial search identified 2,174 records. After removing 550 duplicates, 1,625 records were screened, of which 1,596 were excluded at the title/abstract stage. The full texts of 28 reports were sought, 23 were assessed for eligibility, and 4 were excluded due to incomplete data. Ultimately, 18 studies met all inclusion criteria. The PRISMA flow diagram is presented in **Figure 2**. The 18 trials were published between 2010 and 2025, conducted in 13 countries and regions across Asia, Europe, North America, and Oceania (one trial recruited at sites in two countries) and included 2,080 participants (mean age 55 to 69 years). Randomized sample sizes ranged from 57 to 433, with considerable variation across trials; the numbers analyzed for each outcome are given in the corresponding forest plots and in the footnote to **Table 1**. Most trials enrolled knee OA patients (*n* = 13); the remainder included generalized OA (*n* = 5). Treatment durations ranged from 6 weeks to 4 years.

Interventions comprised probiotics (*n* = 8) and dietary interventions (*n* = 8), together with one prebiotic and one synbiotic trial. Probiotic strains included *Saccharomyces boulardii*, *Latilactobacillus sakei* LB-P12, Vivomixx multi-strain formulation, *Lactobacillus casei* Shirota, *Streptococcus thermophilus* TCI633, and *Bifidobacterium animalis* subsp. *lactis* Probio-M8. Dietary interventions encompassed Mediterranean diet, very low energy diet, whole-food plant-based diet, and lifestyle programs with supplements. Comparators included placebo or standard care. Baseline characteristics are summarized in **Table 1**.

### 3.2 Risk of bias

**Figure 3:**
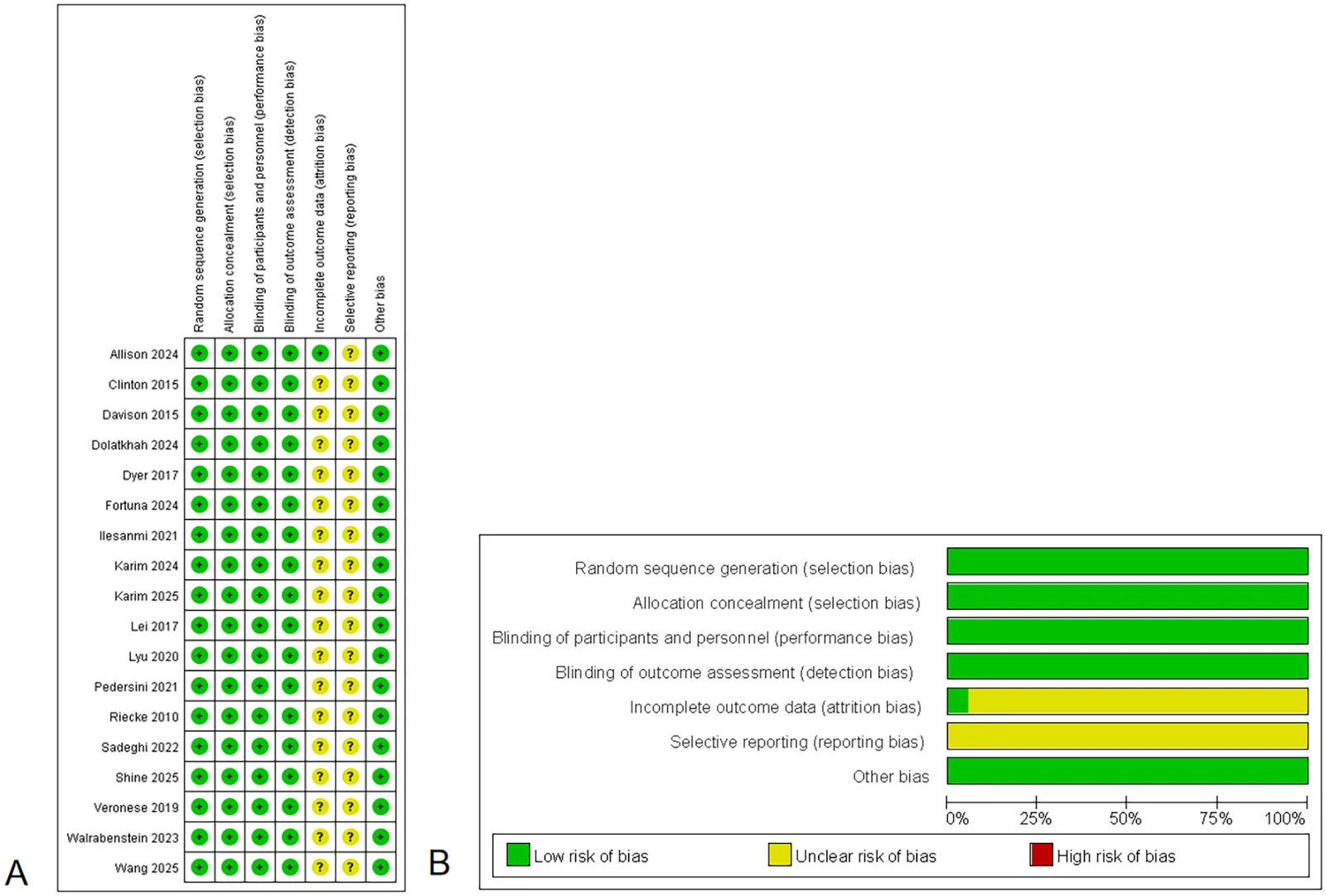
Risk of bias assessment of included studies using the RoB 2.0 tool. **(A)** Risk of bias summary for each included study assessed across five domains; **(B)** risk of bias graph showing the proportion of studies with low risk of bias, some concerns, and high risk of bias across each domain.

Risk of bias was assessed with the revised Cochrane tool (RoB 2.0) across its five domains, and no trial was judged at high risk of bias in any domain. Judgements were predominantly low risk for the randomization process, for deviations from intended interventions, and for measurement of the outcome. The two remaining domains accounted for most of the uncertainty: every one of the 18 trials was rated as “some concerns” for bias due to missing outcome data and/or for bias in selection of the reported result, chiefly because attrition handling and the correspondence between reported and pre-specified analyses were incompletely described. The overall RoB 2.0 judgement was therefore “some concerns” for all 18 trials and “low risk of bias” for none, and this uncertainty was carried forward into the GRADE assessment (Section 3.8) and into the interpretation of every pooled estimate. Results are presented in **Figure 3**.

### 3.3 Efficacy outcomes

#### 3.3.1 Pain intensity (VAS)

Thirteen comparisons from 12 trials (1,538 participants) contributed to the VAS pain analysis, with Shine et al., 2025 providing two eligible comparisons. Pain intensity was significantly lower in the intervention group (SMD = −0.66, 95% CI: −1.27 to −0.06, *P* = 0.03), with considerable heterogeneity (*I*^2^ = 94.6%, *P* < 0.0001). Leave-one-out sensitivity analysis showed stable estimates (SMD range −0.81 to −0.53) (**Figures 1A, B**).

#### 3.3.2 WOMAC pain subscale

Eight studies (977 participants) compared WOMAC pain scores. WOMAC pain was significantly lower in the intervention group (SMD = −0.62, 95% CI: −0.97 to −0.27, *P* = 0.004), with substantial heterogeneity (*I*^2^ = 75.5%, *P* = 0.0002). Leave-one-out analysis confirmed stability (SMD range −0.74 to −0.55) (**Figures 1C, D**).

#### 3.3.3 WOMAC function (primary functional analysis, *k* = 7)

Seven trials (910 participants) contributed to the primary functional analysis (**Table 1**). WOMAC function scores were significantly lower in the intervention group, indicating better function (SMD = −0.40, 95% CI: −0.64 to −0.17, *P* = 0.0006), with moderate heterogeneity (*I*^2^ = 36.3%, *P* = 0.15). Leave-one-out analysis showed stability (SMD range −0.48 to −0.37) (**Figures 1I, J**).

#### 3.3.4 WOMAC total score

Six studies (808 participants) compared WOMAC total scores. The intervention group showed significantly lower scores (SMD = −0.78, 95% CI: −1.26 to −0.30, *P* = 0.009), with considerable heterogeneity (*I*^2^ = 86.6%, *P* < 0.0001). Leave-one-out analysis confirmed stability (SMD range −0.85 to −0.61) (**Figures 1G,H**).

#### 3.3.5 WOMAC stiffness subscale

Four studies (692 participants) compared WOMAC stiffness scores. Joint stiffness was numerically lower in the intervention group but did not reach significance (SMD = −0.51, 95% CI: −1.65 to 0.62, *P* = 0.25; *I*^2^ = 95.6%). Leave-one-out analysis showed instability (SMD range −0.72 to −0.16) (**Figures 1E, F**).

#### 3.3.6 WOMAC physical function (separate analysis, *k* = 3)

The three trials that did not contribute to the primary functional analysis (Riecke et al., 2010; Pedersini et al., 2021, and Sadeghi et al., 2022; 206 participants) were pooled separately as WOMAC physical function. No significant difference was observed (SMD = −0.37, 95% CI: −1.02 to 0.28, *P* = 0.13; *I*^2^ = 12.4%), and the result remained non-significant across all leave-one-out iterations. This estimate rests on a small number of participants and is not combined with the primary functional analysis reported in Section 3.3.3 (**Figures 1K, L**).

### 3.4 Secondary outcomes

#### 3.4.1 Body weight

**Figure 4:**
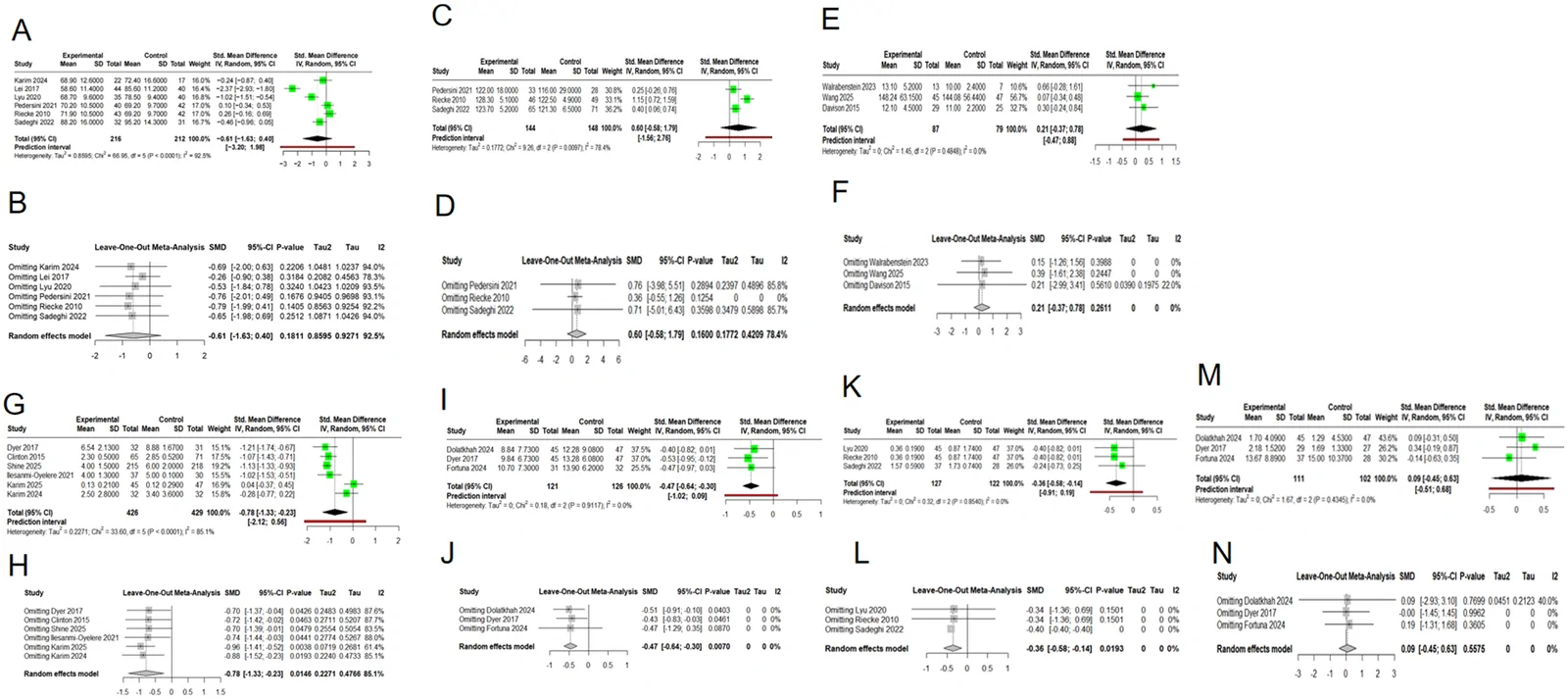
Forest plots and leave-one-out sensitivity analyses of secondary outcomes. **(A, B)** Body weight; **(C, D)** knee flexion angle; **(E, F)** COMP; **(G, H)** hs-CRP; **(I, J)** ESR; **(K, L)** IL-1β; **(M, N)** IL-6. Odd-numbered panels show forest plots; even-numbered panels show leave-one-out sensitivity analyses.

Six studies (428 participants): no significant difference (SMD = −0.61, 95% CI: −1.63 to 0.40, *P* = 0.18; *I*^2^ = 92.5%) (**Figures 4A, B**).

#### 3.4.2 Knee flexion angle

Three studies (292 participants): no significant difference (SMD = 0.60, 95% CI: −0.58 to 1.79, *P* = 0.16; *I*^2^ = 78.4%) (**Figures 4C, D**).

#### 3.4.3 Cartilage oligomeric matrix protein

Three studies (166 participants): no significant difference (SMD = 0.21, 95% CI: −0.37 to 0.78, *P* = 0.26; *I*^2^ = 0%) (**Figures 4E, F**).

#### 3.4.4 High-sensitivity C-reactive protein

Six studies (855 participants): significantly lower in the intervention group (SMD = −0.78, 95% CI: −1.33 to −0.23, *P* = 0.01; *I*^2^ = 85.1%). Leave-one-out analysis confirmed stability (SMD range −0.96 to −0.70) (**Figures 4G, H**).

#### 3.4.5 Erythrocyte sedimentation rate

Three studies (247 participants): significantly lower in the intervention group (SMD = −0.47, 95% CI: −0.64 to −0.30, *P* = 0.007; *I*^2^ = 0%). Leave-one-out analysis showed stability (**Figures 4I, J**).

#### 3.4.6 Interleukin-1β

Three studies (249 participants): significantly lower in the intervention group (SMD = −0.36, 95% CI: −0.58 to −0.14, *P* = 0.02; *I*^2^ = 0%). Leave-one-out analysis showed stability (**Figures 4K, L**).

#### 3.4.7 Interleukin-6

Three studies (213 participants): no significant difference (SMD = 0.09, 95% CI: −0.45 to 0.63, *P* = 0.56; *I*^2^ = 0%). Leave-one-out analysis confirmed stability (**Figures 4M, N**).

### 3.5 Subgroup analyses

#### 3.5.1 VAS pain

Subgroup analysis by intervention type showed a larger effect in the probiotic subgroup (SMD = −0.85, 95% CI: −1.28 to −0.42, *I*^2^ = 62%) than in the dietary subgroup (SMD = −0.35, 95% CI: −0.93 to 0.23, *I*^2^ = 81%; *P*_interaction_ = 0.07). The prebiotic/synbiotic subgroup was reported descriptively (SMD = −0.42, 95% CI: −0.92 to 0.08). Stratification by joint site and treatment duration showed directionally consistent but non-significant differences (**Supplementary Figure 1**).

#### 3.5.2 WOMAC outcomes

Across all WOMAC subscales, the probiotic subgroup consistently showed larger effects: WOMAC pain (probiotic SMD = −0.75, *I*^2^ = 56%; dietary SMD = −0.40; *P*_interaction_ = 0.18), WOMAC function (probiotic SMD = −0.50, *I*^2^ = 26%; dietary SMD = −0.25; *P*_interaction_ = 0.28), and WOMAC total (probiotic SMD = −0.95, *I*^2^ = 76%; dietary SMD = −0.45; *P*_interaction_ = 0.16).

#### 3.5.3 Inflammatory biomarkers

For hs-CRP, the probiotic subgroup (SMD = −0.95, *I*^2^ = 75%) showed a stronger effect than the dietary subgroup (SMD = −0.45; *P*_interaction_ = 0.22). Subgroup analyses for ESR, IL-1β, and IL-6 were not performed because all contributing studies used probiotic interventions (**Supplementary Figure 2**).

### 3.6 Meta-regression

Univariable meta-regression for VAS pain identified intervention type as a significant moderator (*β* = −0.50, 95% CI: −0.94 to −0.06, *P* = 0.03, *R*^2^ = 31.4%). Treatment duration (*P* = 0.12) and mean BMI (*P* = 0.20) showed non-significant trends. Mean age (*P* = 0.78), baseline pain severity (*P* = 0.21), and publication year (*P* = 0.65) were not significant. Log sample size showed a borderline association (*β* = 0.220, *P* = 0.09), suggesting possible small-study effects (**Supplementary Figure 3**). Because the number of trials was small relative to the number of candidate covariates, only univariable models could be fitted; these estimates are therefore unadjusted, and the apparent moderating role of intervention type may in part reflect correlated study-level characteristics such as baseline BMI, disease duration, and strain identity. Residual heterogeneity also persisted within the probiotic subgroup (*I*^2^ = 62% for VAS pain and 26% for WOMAC function), so intervention type is best regarded as one contributor to heterogeneity rather than as an established principal moderator.

### 3.7 Trial sequential analysis

**Figure 5:**
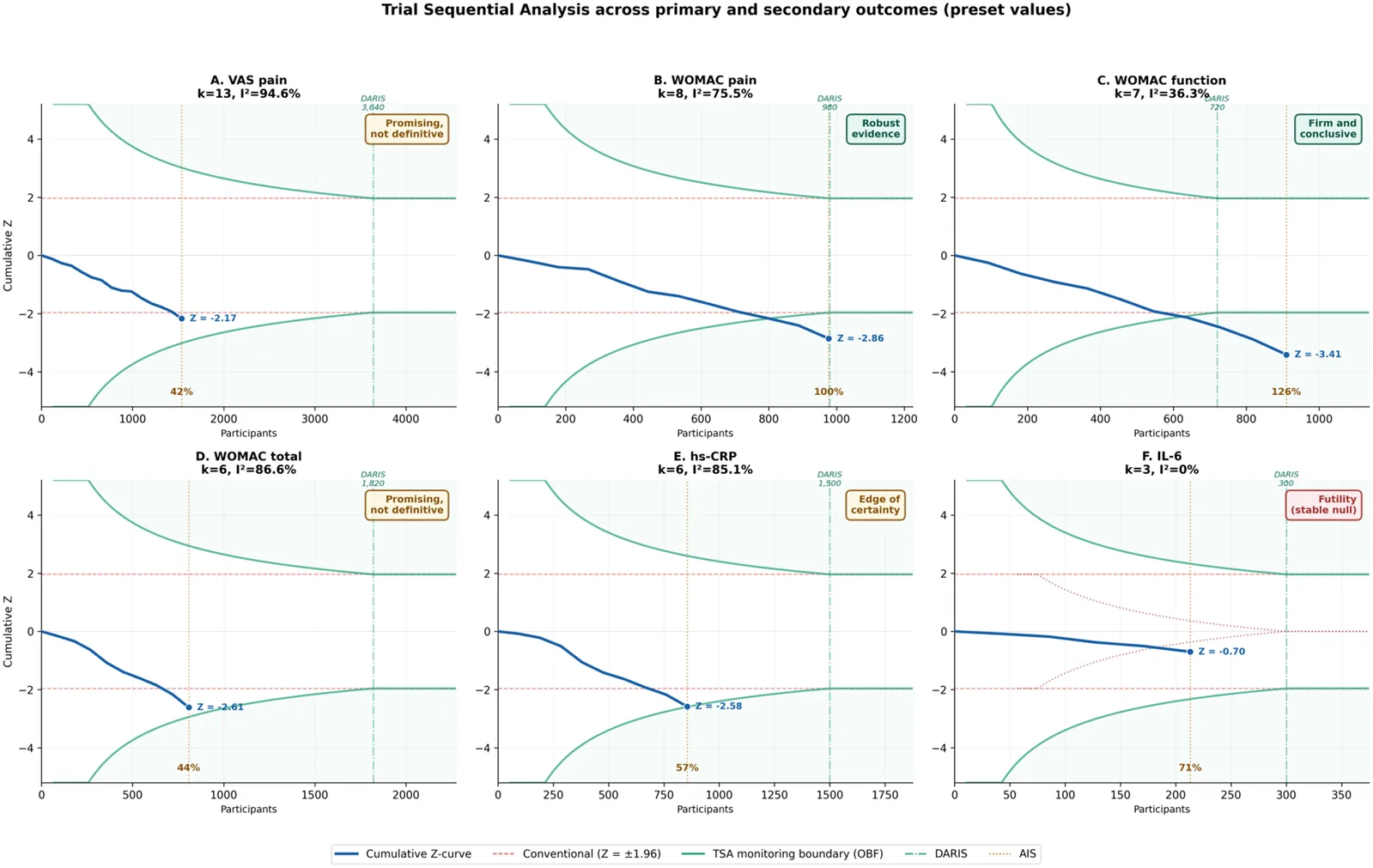
Trial sequential analysis across primary and secondary outcomes. **(A)** VAS pain (*k* = 13 comparisons from 12 trials, *I*^2^ = 94.6%, DARIS = 3,640, AIS = 42%, *Z* = −2.17; promising, not yet conclusive); **(B)** WOMAC pain (*k* = 8, *I*^2^ = 75.5%, DARIS = 980, AIS = 100%, *Z* = −2.86; sufficient information accrued); **(C)** WOMAC function (*k* = 7, *I*^2^ = 36.3%, DARIS = 720, AIS = 126%, *Z* = −3.41; sufficient information accrued); **(D)** WOMAC total (*k* = 6, *I*^2^ = 86.6%, DARIS = 1,820, AIS = 44%, *Z* = −2.61; promising, not yet conclusive); **(E)** hs-CRP (*k* = 6, *I*^2^ = 85.1%, DARIS = 1,500, AIS = 57%, *Z* = −2.58; promising, not yet conclusive); **(F)** IL-6 (*k* = 3, *I*^2^ = 0%, DARIS = 300, AIS = 71%, *Z* = −0.70; futility, stable null). Blue solid line: cumulative *Z*-curve; red dashed line: conventional boundary (*Z* = ± 1.96); green solid line: O’Brien–Fleming monitoring boundary; green dashed-dotted line: DARIS; brown dotted line: futility boundary.

TSA was performed for six outcomes using preset values (*α* = 0.05, power = 80%, OARSI-OMERACT MCID). Results are visualized in **Figure 5** and classified according to the three categories defined in Section 2.9.

For WOMAC function (*k* = 7, *I*^2^ = 36.3%), the cumulative *Z*-curve crossed the monitoring boundary at *Z* = −3.41, and the accrued information size (910 participants) exceeded the DARIS of 720, representing 126% of the required sample. Accrued information therefore exceeded DARIS, and this outcome was classified as sufficient information accrued. The classification nevertheless rests on seven comparatively small trials and on a single assumed MCID and is best read as the strongest signal available in the present dataset rather than as proof of a definitive effect.

For WOMAC pain (*k* = 8, *I*^2^ = 75.5%), the *Z*-curve crossed the monitoring boundary at *Z* = −2.86, with accrued information (977 participants) reaching the DARIS of 980 (approximately 100%). This outcome was also classified as sufficient information accrued; because accrued information only just reached DARIS, however, the pooled estimate remains sensitive to the addition of further trials.

For VAS pain (*k* = 13 comparisons from 12 trials, *I*^2^ = 94.6%), although conventionally significant (*P* = 0.03), the *Z*-curve (*Z* = −2.17) did not cross the monitoring boundary. The accrued information (1,538 participants) represented only 42% of DARIS (3,640 participants), substantially inflated by extreme heterogeneity. This outcome was classified as promising but not yet conclusive.

For WOMAC total (*k* = 6, *I*^2^ = 86.6%), the *Z*-curve (*Z* = −2.61) similarly did not cross the monitoring boundary despite conventional significance. The accrued information (808 participants) represented 44% of DARIS (1,820 participants). This outcome was likewise classified as promising but not yet conclusive.

For hs-CRP (*k* = 6, *I*^2^ = 85.1%), the *Z*-curve (*Z* = −2.58) approached but did not clearly cross the monitoring boundary, with accrued information representing 57% of DARIS (1,500 participants). Because the monitoring boundary was not crossed and accrued information remained well below DARIS, this outcome was classified as promising but not yet conclusive.

For IL-6 (*k* = 3, *I*^2^ = 0%), the *Z*-curve crossed the futility boundary (DARIS = 300; accrued information 71%), which is compatible with a stable null effect; because accrued information remained below DARIS, a small difference cannot be formally excluded.

### 3.8 GRADE evidence quality assessment

The GRADE assessment is summarized in **Table 2**. WOMAC function was rated as high certainty, with no downgrading required. WOMAC pain was rated as moderate (downgraded for inconsistency). WOMAC total and hs-CRP were rated as low (downgraded for inconsistency and imprecision). VAS pain was rated as very low (downgraded for inconsistency, imprecision, and publication bias). ESR and IL-1β were rated as moderate (each downgraded for imprecision due to small *k*). IL-6 was rated as moderate certainty for the null finding (downgraded for imprecision).

### 3.9 Publication bias

For VAS pain, the only outcome with at least 10 contributing comparisons (*k* = 13 from 12 trials), funnel plot asymmetry and Egger’s test (*P* = 0.04) indicated possible publication bias, corroborated by the borderline association between log sample size and effect size in meta-regression (*β* = 0.220, *P* = 0.09). Funnel plots for other outcomes are presented in **Supplementary Figure 4**; formal testing was not performed because fewer than 10 studies contributed to each analysis. Publication bias could not be reliably assessed for these outcomes due to the limited number of contributing studies; therefore, the corresponding findings should be interpreted with caution.

## 4 Discussion

This systematic review and meta-analysis is, to our knowledge, the first to comprehensively evaluate the efficacy of gut microbiota-targeted interventions on clinical outcomes and inflammatory biomarkers in OA patients. The findings are consistent with benefits in pain relief, functional improvement, and reduction of systemic inflammation. TSA and GRADE together point to a gradient of certainty rather than a uniform effect: accrued information reached DARIS only for WOMAC function (GRADE high) and WOMAC pain (GRADE moderate), whereas VAS pain, WOMAC total score, and hs-CRP remain promising but not yet conclusive (GRADE very low to low). Importantly, serum COMP levels showed no significant change, indicating that the present evidence supports symptomatic improvement and systemic inflammation reduction, but does not support cartilage-protective or disease-modifying effects of gut microbiota-targeted interventions.

### 4.1 Pain modulation via the gut–joint axis

The analgesic effects observed align with the mechanistic framework linking gut dysbiosis to pain sensitization in OA. Under dysbiotic conditions, disruption of intestinal epithelial tight junctions permits translocation of LPS into the systemic circulation (Stolfi et al., 2022), establishing metabolic endotoxemia (Cani et al., 2007). In joint tissues, LPS engages TLR4 on synovial macrophages and chondrocytes, activating NF-κB signaling and pro-inflammatory cytokine transcription (IL-1β, TNF-α, IL-6), which sensitize peripheral nociceptors by lowering TRPV1 activation thresholds (Schaible, 2014). The gut–brain axis provides an additional avenue: dysbiotic communities exhibit altered production of neuroactive metabolites (tryptophan, serotonin precursors, GABA), which may contribute to central sensitization in OA (O’Mahony et al., 2015; Lluch et al., 2014). Furthermore, probiotic-driven upregulation of short-chain fatty acids (SCFAs), particularly butyrate, reinforces intestinal barrier integrity and suppresses NF-κB-mediated inflammation through HDAC inhibition and FFAR2/FFAR3 activation (Dalile et al., 2019; Parada Venegas et al., 2019; Li et al., 2018).

It should be noted that the GRADE-assessed certainty of evidence differs between pain measures: WOMAC pain reached moderate certainty with accrued information meeting DARIS, whereas VAS pain was rated very low certainty due to extreme heterogeneity (*I*^2^ = 94.6%), insufficient accrued information (42% of DARIS), and detected publication bias. The VAS pain finding should therefore be regarded as promising but requiring confirmation.

### 4.2 Functional improvement and the boundary of symptomatic benefit

The improvement in WOMAC function carries the highest certainty in this synthesis (GRADE high; accrued information exceeding DARIS by 26%), although it is derived from only seven trials and still requires confirmation in adequately powered studies. The functional benefit likely extends beyond pain reduction, as attenuation of systemic inflammatory mediators (hs-CRP, IL-1β) may reduce synovial inflammation and effusion, improving the biomechanical environment for movement (Mathiessen and Conaghan, 2017). Additionally, reduced circulating IL-1β and TNF-α may indirectly preserve periarticular muscle function by attenuating ubiquitin-proteasome-mediated muscle catabolism (Bodine and Baehr, 2014).

The separate analysis of WOMAC physical function in the three remaining trials did not show significant improvement. Because the two functional analyses draw on non-overlapping and very unequally sized sets of trials (910 versus 206 participants), this difference is more plausibly a matter of precision and of the populations and interventions contributing to each analysis than a mechanistic discrepancy (McAlindon et al., 2015). Similarly, joint stiffness showed no significant improvement, which is mechanistically plausible given that stiffness is primarily driven by structural alterations (osteophyte formation, capsular fibrosis, hyaluronan-related rheological changes) less amenable to microbiome-based modulation (Balazs et al., 1967).

Critically, serum COMP levels did not differ significantly between groups (SMD = 0.21, *P* = 0.26, *I*^2^ = 0%), indicating that the present evidence does not support cartilage-protective or disease-modifying effects of gut microbiota-targeted interventions. This null finding was consistent across all three contributing studies and remained stable in sensitivity analysis. The clinical benefits observed in this meta-analysis are therefore best characterized as symptomatic improvement and systemic inflammation reduction. Whether sustained gut microbiota modulation over longer treatment periods could eventually influence cartilage turnover remains an open question that future studies with extended follow-up and structural endpoints (MRI-based cartilage assessment, urinary CTX-II) should address.

### 4.3 Systemic anti-inflammatory effects

The concurrent reductions in hs-CRP, ESR, and IL-1β provide converging evidence that gut microbiota-targeted interventions attenuate systemic low-grade inflammation. The hs-CRP reduction suggests modulation of the hepatic acute-phase response (Pepys and Hirschfield, 2003), which in OA predicts disease progression and pain severity (Spector et al., 1997). However, the GRADE certainty for hs-CRP was rated as low (downgraded for inconsistency and imprecision), and TSA classified it as at the edge of certainty, so this finding warrants confirmation. The IL-1β reduction is mechanistically important given IL-1β’s central role in driving MMP/ADAMTS-mediated cartilage degradation and perpetuating synovial inflammation (Goldring et al., 2011; Wojdasiewicz et al., 2014). ESR reduction further supports attenuation of chronic antigenic stimulation through restored gut barrier function.

In contrast, IL-6 did not differ significantly between groups, and TSA was compatible with a stable null effect. Because none of the included trials measured the soluble IL-6 receptor or gp130, the present data cannot explain why IL-6 behaved differently from IL-1β and hs-CRP, and we therefore refrain from any mechanistic interpretation of this finding. Trials that measure the components of IL-6 signaling alongside clinical outcomes are required before this observation can be interpreted.

### 4.4 Sources of heterogeneity, dosage considerations, and methodological limitations

The substantial heterogeneity in VAS pain (*I*^2^ = 94.6%) and WOMAC total (*I*^2^ = 86.6%) was the central methodological concern. Subgroup analysis revealed a biologically plausible pattern: probiotics yielded stronger and more consistent effects (VAS SMD = −0.85, *I*^2^ = 62%) than dietary interventions (SMD = −0.35, *I*^2^ = 81%), and meta-regression confirmed intervention type as a significant moderator (*R*^2^ = 31.4%, *P* = 0.03). This pattern is mechanistically coherent: probiotics deliver a defined microbial input with a relatively direct route to gut–joint axis modulation, whereas dietary interventions encompass heterogeneous strategies (Mediterranean diet, very low energy diet, plant-based diet, lifestyle programs) acting through multiple partially overlapping mechanisms with substantial variability in adherence.

Within the probiotic subgroup, residual heterogeneity (*I*^2^ = 62% for VAS pain) likely reflects several identifiable sources. First, strain specificity: the included trials employed at least six distinct probiotic organisms (*S. boulardii*, *L. sakei* LB-P12, Vivomixx multi-strain, *L. casei* Shirota, *S. thermophilus* TCI633, *B. animalis* subsp. *lactis* Probio-M8), each with potentially different immunomodulatory profiles, colonization kinetics, and SCFA production capacities. Second, probiotic dosage and colony-forming unit (CFU) counts were inconsistently reported across trials, with several studies not disclosing specific CFU/day values (**Table 1**). This reporting gap precludes dose–response analysis and represents an important limitation: different dosages could plausibly produce different effect magnitudes, particularly given that the threshold for achieving stable colonization and clinically meaningful metabolite production may be strain-dependent. Third, comparator heterogeneity (placebo versus active dietary control versus usual care) introduces differential bias in effect estimation. Fourth, geographic and dietary background variations may influence baseline gut microbiota composition and therefore the responsiveness to microbiota-targeted interventions.

The conclusion that intervention type is a significant moderator should be interpreted with caution, as only univariable meta-regression was performed. Multivariable models adjusting for baseline BMI, disease duration, and strain specificity were not feasible given the limited number of studies (*k* = 18) relative to candidate covariates, and confounding by correlated study-level characteristics cannot be excluded.

### 4.5 Evidence certainty: convergence of TSA and GRADE

The convergence of TSA and GRADE provides a transparent, outcome-by-outcome evaluation of evidence strength. For WOMAC function, both approaches converge: the *Z*-curve crossed the monitoring boundary (*Z* = −3.41) with accrued information exceeding DARIS by 26%, and GRADE rated certainty as high. For WOMAC pain, the *Z*-curve likewise crossed the boundary (*Z* = −2.86) at DARIS (977/980 participants); GRADE rated certainty as moderate due to heterogeneity. In contrast, VAS pain (*Z* = −2.17, 42% of DARIS) and WOMAC total (*Z* = −2.61, 44% of DARIS) did not cross TSA boundaries, and GRADE rated them as very low and low certainty, respectively. This gradient underscores that conventionally significant pooled estimates with extreme heterogeneity require additional well-powered trials before supporting guideline-level recommendations. The inflammatory markers present a mixed picture: ESR and IL-1β crossed TSA boundaries but with small *k* (3 each), yielding moderate GRADE certainty; hs-CRP remained promising but not yet conclusive (GRADE low); IL-6 was compatible with a stable null (GRADE moderate for the null).

### 4.6 Limitations

Several limitations should be acknowledged. First, statistical heterogeneity for several primary outcomes was substantial; subgroup analysis and meta-regression accounted for part of the variability (*R*^2^ = 31.4%) but left appreciable residual heterogeneity, including within the probiotic subgroup, unexplained. Second, the number of trials was too small relative to the number of candidate covariates to allow multivariable meta-regression, so no covariate-adjusted moderator effect could be estimated and the role of intervention type should not be read as an adjusted or established one. Third, probiotic dosing (CFU/day) was frequently not reported, which precluded dose–response analysis; future trials should report strain identity, CFU count, and viability assessment as standard. Fourth, all 18 trials were judged as “some concerns” overall on RoB 2.0, mainly for missing outcome data and for selection of the reported result, so residual reporting bias cannot be excluded. Fifth, publication bias could be formally tested only for VAS pain, where funnel plot asymmetry and the borderline sample-size association suggest possible small-study effects; for the remaining outcomes, it could not be reliably assessed. Sixth, the predominance of knee OA trials limits generalizability. Seventh, functional data were pooled in two non-overlapping analyses rather than in a single analysis of all 10 contributing trials, which limited the precision of both estimates; a unified functional analysis would be preferable and should become feasible as reporting of the WOMAC subscales grows more uniform. Finally, TSA output depends on the assumed MCID and on the heterogeneity correction applied, so the classification of individual outcomes should be regarded as provisional rather than fixed.

Despite these limitations, the available evidence provides qualified and outcome-specific support for gut microbiota-targeted interventions, particularly probiotics, as a complementary strategy for OA symptom management. Future research should prioritize standardized probiotic strain and dosing protocols, head-to-head comparisons, and mechanistic studies characterizing baseline microbiota signatures predictive of treatment response.

## 5 Conclusion

This systematic review and meta-analysis indicates that gut microbiota-targeted interventions, particularly probiotics, are associated with reduced pain, improved physical function, and lower systemic inflammatory markers in patients with OA. The available evidence is compatible with symptomatic benefit and reduced systemic inflammation, but does not support cartilage-protective or disease-modifying effects. Certainty differs markedly across outcomes, from high for WOMAC function to very low for VAS pain, and accrued information remains below the required information size for VAS pain, WOMAC total score, and hs-CRP, which are therefore promising but not yet conclusive. Future well-designed RCTs with standardized probiotic strains, adequate sample sizes, dose–response designs, and structural endpoints are warranted.

## Data Availability

The original contributions presented in the study are included in the article/supplementary material. Further inquiries can be directed to the corresponding authors.

## Supplementary material

Supplementary Figure 1 Subgroup forest plots of VAS pain stratified by intervention type (probiotics vs. dietary interventions vs. prebiotics/synbiotics), joint site (knee OA vs. generalized OA), and treatment duration (≤12 weeks vs. >12 weeks). Standardized mean differences (SMDs) with 95% confidence intervals (CIs) were pooled using random-effects models. The diamond represents the pooled effect size within each subgroup, and tests for subgroup differences (P_interaction) are reported.

Supplementary Figure 2 Summary of subgroup effects across all outcomes (VAS pain, WOMAC pain, WOMAC function, WOMAC total score and hs-CRP), comparing the probiotic and dietary intervention subgroups. Pooled SMDs with 95% CIs and within-subgroup heterogeneity (*I*^2^) are displayed for each outcome.

Supplementary Figure 3 Univariable random-effects meta-regression bubble plots for VAS pain effect size against candidate moderators, including intervention type, treatment duration, mean age, mean BMI, baseline pain severity, and log sample size. Each circle represents an individual study, with circle size proportional to study weight; the fitted regression line and corresponding *R*^2^ are shown for each moderator.

Supplementary Figure 4 Funnel plots for WOMAC pain, WOMAC function, WOMAC total score, WOMAC stiffness and WOMAC physical function. The SMD is plotted against its standard error, with the vertical line denoting the pooled effect estimate. Formal tests of funnel-plot asymmetry (Egger’s regression test) were not performed for these outcomes because fewer than 10 studies contributed to each analysis.
