# Supplementary material for: Effects of gut microbiota-targeted interventions on pain, physical function, and inflammatory biomarkers in osteoarthritis: a systematic review and meta-analysis of randomized controlled trials
Source: Front Cell Infect Microbiol. 2026 Sep 4;16:1907672. doi: 10.3389/fcimb.2026.1907672 (PMC13585646; doi:10.3389/fcimb.2026.1907672)
Supplement: Supplementary file 1 [file Supplementaryfile1.docx]

**Supplementary Figure S2. Subgroup forest plots by intervention type across primary and key secondary outcomes.** (A) VAS pain (k = 13); (B) WOMAC pain (k = 8); (C) WOMAC physical function (k = 7); (D) WOMAC total score (k = 6); (E) High-sensitivity C-reactive protein (k = 6). Each panel displays individual study effect sizes (squares) and 95% confidence intervals (horizontal lines) within probiotic and dietary subgroups, subgroup pooled estimates (diamonds), and the overall pooled estimate. P_interaction values are shown for each outcome.
