# Supplementary material for: Effects of gut microbiota-targeted interventions on pain, physical function, and inflammatory biomarkers in osteoarthritis: a systematic review and meta-analysis of randomized controlled trials
Source: Front Cell Infect Microbiol. 2026 Sep 4;16:1907672. doi: 10.3389/fcimb.2026.1907672 (PMC13585646; doi:10.3389/fcimb.2026.1907672)
Supplement: Supplementary file 2 [file Supplementaryfile2.docx]

**Supplementary Figure S3. Summary heatmap of subgroup effect sizes and evidence certainty across all outcomes.** Rows represent outcomes; columns represent the overall pooled estimate, probiotic subgroup, and dietary subgroup. Cell color intensity reflects the magnitude of the standardized mean difference (deeper color = larger beneficial effect). Each cell displays the SMD with significance markers (* P < 0.05, ** P < 0.01, *** P < 0.001, ns = non-significant), 95% confidence interval, and I² statistic (color-coded: green < 50%, amber 50–75%, red > 75%). The P_interaction column shows the test for subgroup differences, and the TSA column shows the Trial Sequential Analysis conclusion for each outcome.
