# Supplementary material for: Effects of gut microbiota-targeted interventions on pain, physical function, and inflammatory biomarkers in osteoarthritis: a systematic review and meta-analysis of randomized controlled trials
Source: Front Cell Infect Microbiol. 2026 Sep 4;16:1907672. doi: 10.3389/fcimb.2026.1907672 (PMC13585646; doi:10.3389/fcimb.2026.1907672)
Supplement: Supplementary file 3 [file Supplementaryfile3.docx]

**Supplementary Figure S4**. Meta-regression bubble plot: treatment duration versus effect size for VAS pain. Each bubble represents one study; bubble size is proportional to inverse-variance weight. Purple circles represent probiotic interventions (k = 8); amber circles represent dietary interventions (k = 4); green circles represent prebiotic/synbiotic interventions (k = 1). The dashed line indicates the fitted univariable random-effects meta-regression line (β = −0.030 per week, 95% CI: −0.068 to 0.008, P = 0.12; R²_analog = 9.8%). The shaded band represents the 95% confidence interval for the regression line.
