# Supplementary material for: Effects of gut microbiota-targeted interventions on pain, physical function, and inflammatory biomarkers in osteoarthritis: a systematic review and meta-analysis of randomized controlled trials
Source: Front Cell Infect Microbiol. 2026 Sep 4;16:1907672. doi: 10.3389/fcimb.2026.1907672 (PMC13585646; doi:10.3389/fcimb.2026.1907672)
Supplement: Supplementary file 4 [file Supplementaryfile4.docx]

**Supplementary Figure S5. Funnel plots for the assessment of publication bias across primary outcomes. (A) Visual Analogue Scale (VAS); (B) WOMAC pain subscale; (C) WOMAC function subscale; (D) WOMAC total score; (E) WOMAC stiffness subscale; (F) WOMAC physical function subscale.**
